# A new fine‐grained method for automated visual analysis of herbarium specimens: A case study for phenological data extraction

**DOI:** 10.1002/aps3.11368

**Published:** 2020-07-01

**Authors:** Hervé Goëau, Adán Mora‐Fallas, Julien Champ, Natalie L. Rossington Love, Susan J. Mazer, Erick Mata‐Montero, Alexis Joly, Pierre Bonnet

**Affiliations:** ^1^ AMAP University of Montpellier CIRAD CNRS INRAE IRD Montpellier France; ^2^ CIRAD UMR AMAP Montpellier France; ^3^ School of Computing Costa Rica Institute of Technology Cartago Costa Rica; ^4^ Institut national de recherche en informatique et en automatique (INRIA) Sophia‐Antipolis, ZENITH team Laboratory of Informatics Robotics and Microelectronics–Joint Research Unit, 34095 Montpellier CEDEX 5 France; ^5^ Department of Ecology, Evolution, and Marine Biology University of California, Santa Barbara Santa Barbara California 93106 USA

**Keywords:** automated regional segmentation, deep learning, herbarium data, natural history collections, phenological stage annotation, phenophase, regional convolutional neural network, visual data classification

## Abstract

**Premise:**

Herbarium specimens represent an outstanding source of material with which to study plant phenological changes in response to climate change. The fine‐scale phenological annotation of such specimens is nevertheless highly time consuming and requires substantial human investment and expertise, which are difficult to rapidly mobilize.

**Methods:**

We trained and evaluated new deep learning models to automate the detection, segmentation, and classification of four reproductive structures of *Streptanthus tortuosus* (flower buds, flowers, immature fruits, and mature fruits). We used a training data set of 21 digitized herbarium sheets for which the position and outlines of 1036 reproductive structures were annotated manually. We adjusted the hyperparameters of a *mask R‐CNN* (regional convolutional neural network) to this specific task and evaluated the resulting trained models for their ability to count reproductive structures and estimate their size.

**Results:**

The main outcome of our study is that the performance of detection and segmentation can vary significantly with: (i) the type of annotations used for training, (ii) the type of reproductive structures, and (iii) the size of the reproductive structures. In the case of *Streptanthus tortuosus*, the method can provide quite accurate estimates (77.9% of cases) of the number of reproductive structures, which is better estimated for flowers than for immature fruits and buds. The size estimation results are also encouraging, showing a difference of only a few millimeters between the predicted and actual sizes of buds and flowers.

**Discussion:**

This method has great potential for automating the analysis of reproductive structures in high‐resolution images of herbarium sheets. Deeper investigations regarding the taxonomic scalability of this approach and its potential improvement will be conducted in future work.

Herbaria represent major and unique resources for the study of plant phenology over time and space (Willis et al., 2017a; Yost et al., 2018). In particular, they provide the only tangible, long‐term evidence of sustained changes in plant phenology from the beginning of the 19th century to the present. Global awareness of climate change has led to a renewed interest in herbarium‐derived data for the study of climate‐induced shifts in the seasonal cycles of plants (Davis et al., 2015; Soltis et al., 2018; Lang et al., 2019). Several recent herbarium‐based studies have detected changes in the flowering times of many taxa over the past century (Primack et al., 2004; Munson and Long, 2017; Pearson, 2019a). An important next step is to scale up such studies, extending them to much larger taxonomic and geographical ranges, to provide more comprehensive analyses of the potential impacts of such shifts on plant–pollinator interactions and on the phenological properties of entire plant communities.

The recent massive digitization of herbarium‐derived information from around the world has enabled a wide variety of scientists to use it for phenological research (Soltis, 2017); however, most studies designed to detect sources of variation in the flowering times of wild species have classified the phenological status of specimens using qualitative categories, such as the presence/absence of flowers, in combination with the day of year of collection to estimate the flowering time of individuals and taxa. This approach has revealed variation among species with respect to the sensitivity of their flowering times to a variety of climatic parameters (Park and Mazer, 2018), but does not control for the notable variation among specimens in their precise phenological status, which may vary from the initiation of flower buds to the production of ripe fruits. Only rare experiments (such as CrowdCurio, Willis et al., 2017b) have been conducted to provide detailed quantitative counts on all specimens of a collection for the purposes of assessing phenological change within and between seasons. The power of this approach has revealed substantial and unexpected variation in phenological sensitivity across the eastern United States (Park et al., 2019).

Quantitative measures of the phenological status of specimens, based on the estimated proportions of buds, flowers, and fruits, are particularly useful for the analysis of species with high numbers of structures in their inflorescences and/or infructescences, such as species of the Asteraceae (see Pearson, 2019b, for an example of the precise phenological status estimation method). In such taxa, quantitative assessments of the phenological status of individual plants may help to improve the explanatory power of pheno‐climatic models, but this approach requires precise annotations (such as recording the number of reproductive structures representing different stages of development) in order to accurately estimate phenological status (cf. Mulder et al., 2017; Love et al., 2019a).

The precise and detailed counting of reproductive structures in herbarium images is rarely conducted and the annotation requires significant time and expertise when performed by human observers (Brenskelle et al., 2020). Given the time required to record this information (such as the number and size of buds, open flowers, spent flowers, and immature/mature fruits) for the tens of millions of specimens already digitized, and the millions yet to come, it will take several decades to obtain this precise information if we rely solely on manual annotations. A few programs have experimented with online collaborative approaches (e.g., Notes from Nature [https://www.zooniverse.org/organizations/md68135/notes‐from‐nature]) for recording detailed phenological information. Other approaches, such as the ones studied by Lorieul et al. (2019), have experimented with the use of deep learning techniques to automate basic annotations, such as the presence/absence of flowers and/or fruits, on a scale of several tens of thousands of herbarium specimens. The promising results reported by Lorieul et al. (2019) led them to focus on the estimation of finer phenological information, such as the precise phenophase of a specimen, which has contributed to open new ways of automatically determining the phenological stage of a specimen using a quantitative metric.

Plant collectors seem to maximize the richness of information of the plant specimens that they collect by collecting samples with the maximum diversity of botanical structures (including reproductive ones). This has several benefits, including (i) facilitating the species identification of the specimens, (ii) providing a richer contribution to the knowledge of the species’ morphological diversity, and (iii) enabling a greater potential use of the specimen in different research questions. This practice can partially explain why Lorieul et al. (2019) reported that, of the 163,233 herbarium specimens sampled from three herbarium collections (temperate to equatorial), 79.4% to 92.7% showed currently reproducing specimens, with a much higher proportion of them displaying flowers than fruits.

The numbers of different classes of reproductive structures (e.g., buds, open flowers, developing ovaries, and fruits) can be used to calculate a phenological index, a quantitative metric of the phenological progression of each specimen based on the proportions of distinct reproductive structures. This metric has been used by Mulder et al. (2017) and Love et al. (2019a). Love et al. (2019a) demonstrated that the inclusion of this metric as an independent variable improves the explanatory power of pheno‐climatic models designed to detect the effect of climate on flowering date, primarily because the metric itself explains a good deal of variance in the date of specimen collection (i.e., phenologically advanced specimens tend to be those collected at relatively late dates in the year).

The large‐scale, precise, rapid, and automated detection and identification of reproductive structures in herbarium specimens would likely greatly increase the use of herbarium collections in climate change studies. To our knowledge, no approach in the scientific literature has attempted to automate locating, segmenting, and counting distinct types of reproductive structures in digitized herbarium specimens. Here, we aim to fill this gap by evaluating the use of a state‐of‐the‐art *instance segmentation* approach, called *mask R‐CNN* (regional convolutional neural network, He et al., 2017), for recording the phenological information of the reproductive structures borne by *Streptanthus tortuosus* Kellogg (Brassicaceae). This species, native to California, is well‐suited for this study due to (i) its relatively small size, which allows for entire plants (or a significant portion of them) to be preserved on a single herbarium sheet; (ii) its strong visual similarity to congeners, facilitating the application of the methods developed here to a larger taxonomic scale (i.e., within the genus); and (iii) the clear phenotypic differences among its distinct reproductive phases, which include closed buds, open flowers, immature fruits, and ripe fruits. The clear differences between developmental stages provide two benefits. First, they promote consistent scoring among human observers, which leads to high‐quality training data sets, and second, they facilitate the discrimination among organ types by machine learning algorithms and the resulting quantitative assessment of each specimen’s phenological status. In addition, the species (as well as the genus as a whole) is of great interest to botanists because it is widely distributed across latitudinal, longitudinal, and elevation gradients. Consequently, it is highly suitable for use in phenoclimatic models designed to detect the effects of extrinsic factors (e.g., climate, soil type) on the onset and duration of flowering (Love et al., 2019a).

Our study was designed to compare the impact of different methodological approaches toward training deep learning models for instance segmentation. In particular, we evaluate the performance of the model based on three types of annotation (points masks, partial masks, and full masks), all performed on the same herbarium data set. The strengths and constraints of each type of annotation are assessed and discussed, and we offer recommendations for the generalization of these methods to other taxonomic groups or to a larger taxonomic scale. Moreover, we evaluate the applicability of the best approach for two concrete analysis tasks: (i) automatically counting the number of each reproductive structure per sheet, and (ii) automatically estimating their average size per sheet.

## METHODS

### Data sets

**FIGURE 1:**
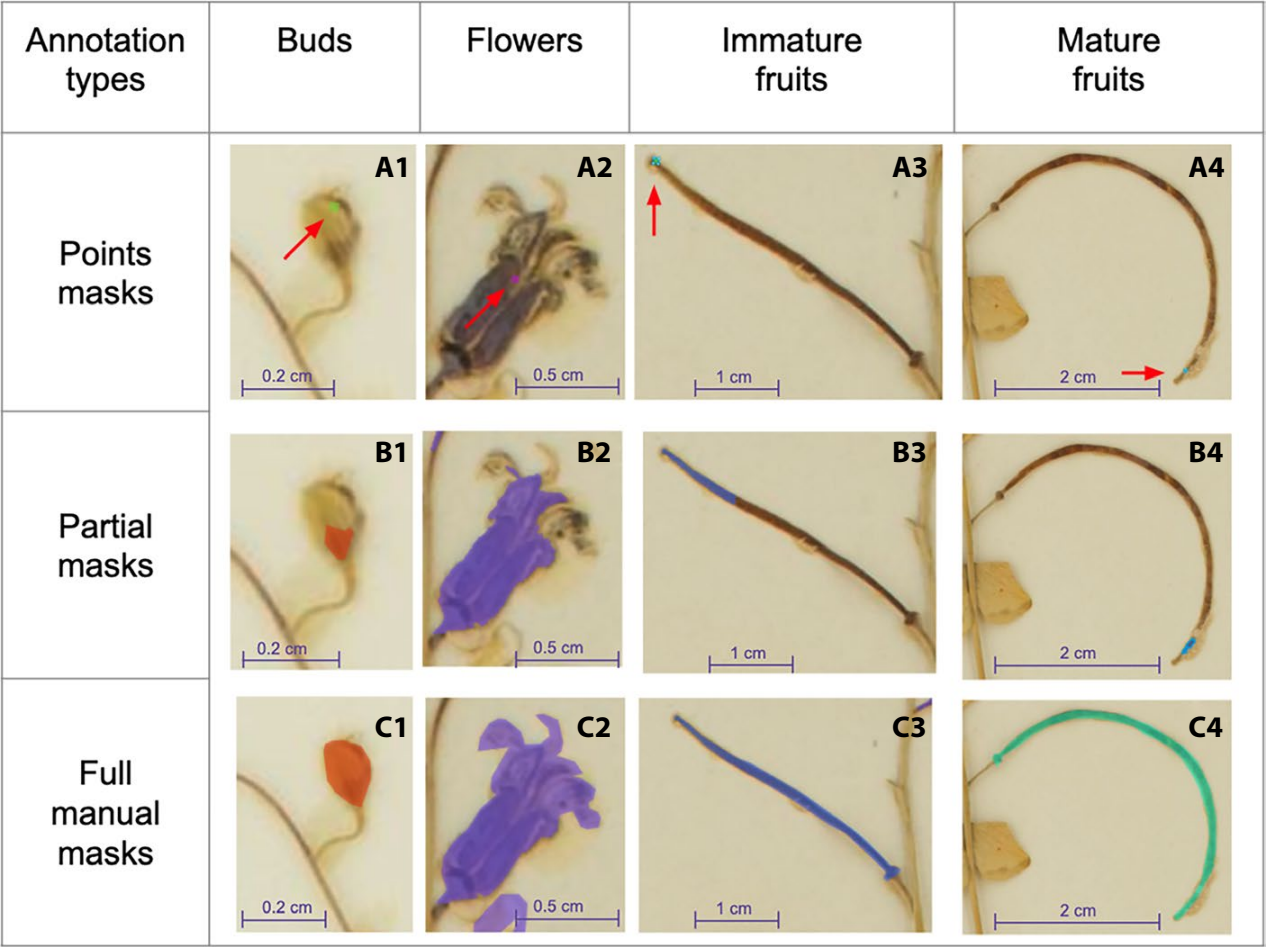
Illustration of the three different types of training data used in this study, for the same four different types of reproductive structures analyzed (i.e., Buds, Flowers, Immature Fruits, Mature Fruits). (A) Points masks. (B) Partial masks. (C) Full manual masks. Red arrows highlight the points masks, which are too small to be easily visible at this scale.

To evaluate the performance of the mask R‐CNN approach on different types of annotations, we produced three different training sets, all of them obtained from the same initial data set of imaged and hand‐scored herbarium specimen sheets produced by Love et al. (2019b). The input images were resized such that the longest edge was 2048 pixels and the shorter one was 1024 pixels in order to have sufficient numbers of pixels comprising small objects such as the buds. These three training data sets are each based on one of three methods for producing annotations of reproductive structures. The first training set (“*PointsMask*”) is based on the protocol designed by Love et al. (2019a) to score and record the number of reproductive structures representing successive developmental stages on imaged herbarium specimens using the plugin Cell Counter (https://imagej.nih.gov/ij/plugins/cell‐counter.html), developed for the image analysis software ImageJ (Abramoff, 2004). It is important to note that this protocol was not developed to produce labeled data for training machine learning algorithms; rather, it was designed for use in manual organ counting. To score each specimen, the annotator (N.L.R.L., S.J.M., and two collaborators) placed a digital colored marker near the distal extremity or at the center of each visible reproductive structure; each type of organ (buds, flowers, immature fruits, mature fruits) was indicated by a distinct marker. The scoring of each specimen was visually checked for accuracy by N.L.R.L., and any incorrectly categorized organs were corrected before using the markers to construct visual masks. For each point, we produced a small square (3 × 3 pixels) mask (i.e., a shape file) close to the top of the recorded reproductive structure (Fig. 1A).

**FIGURE 2:**
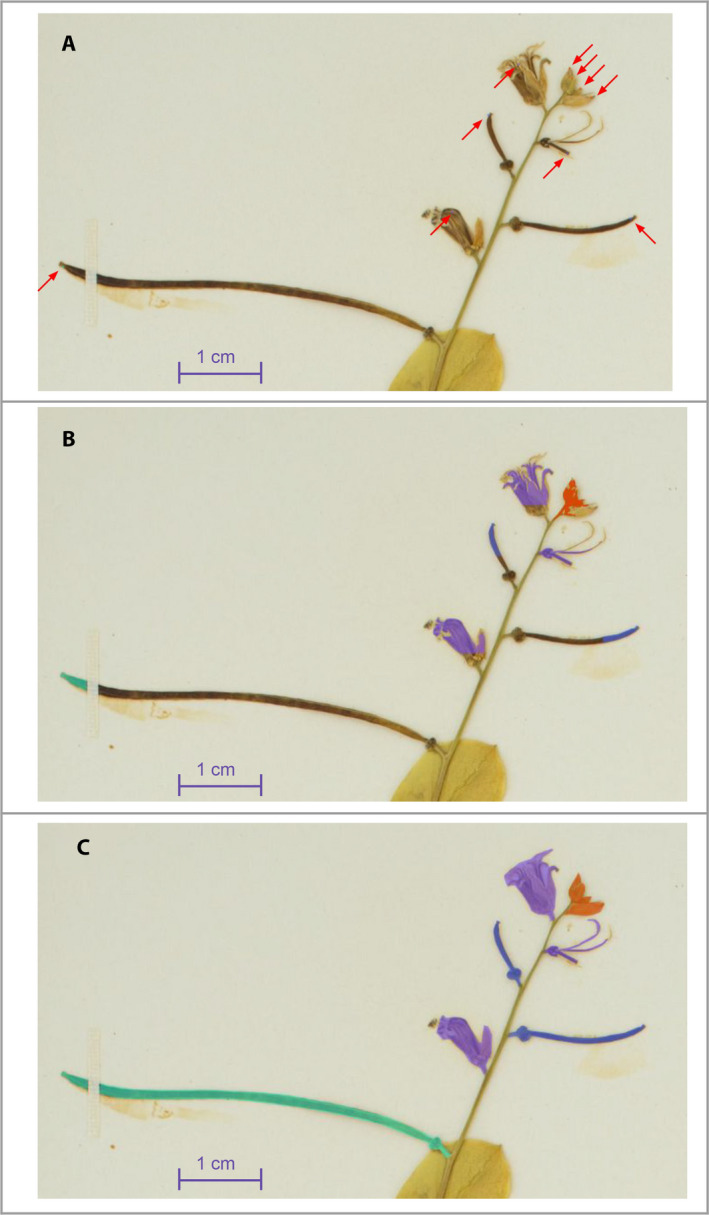
Illustration of the three different types of training data used in this study. (A) Points masks. (B) Partial masks. (C) Full manual masks. The same inflorescence is displayed in A–C to indicate the four different types of reproductive structures analyzed (i.e., Buds in orange, Flowers in purple, Immature Fruits in blue, Mature Fruits in green). Red arrows highlight the points masks, which are too small to be easily visible at this scale.

**FIGURE 3:**
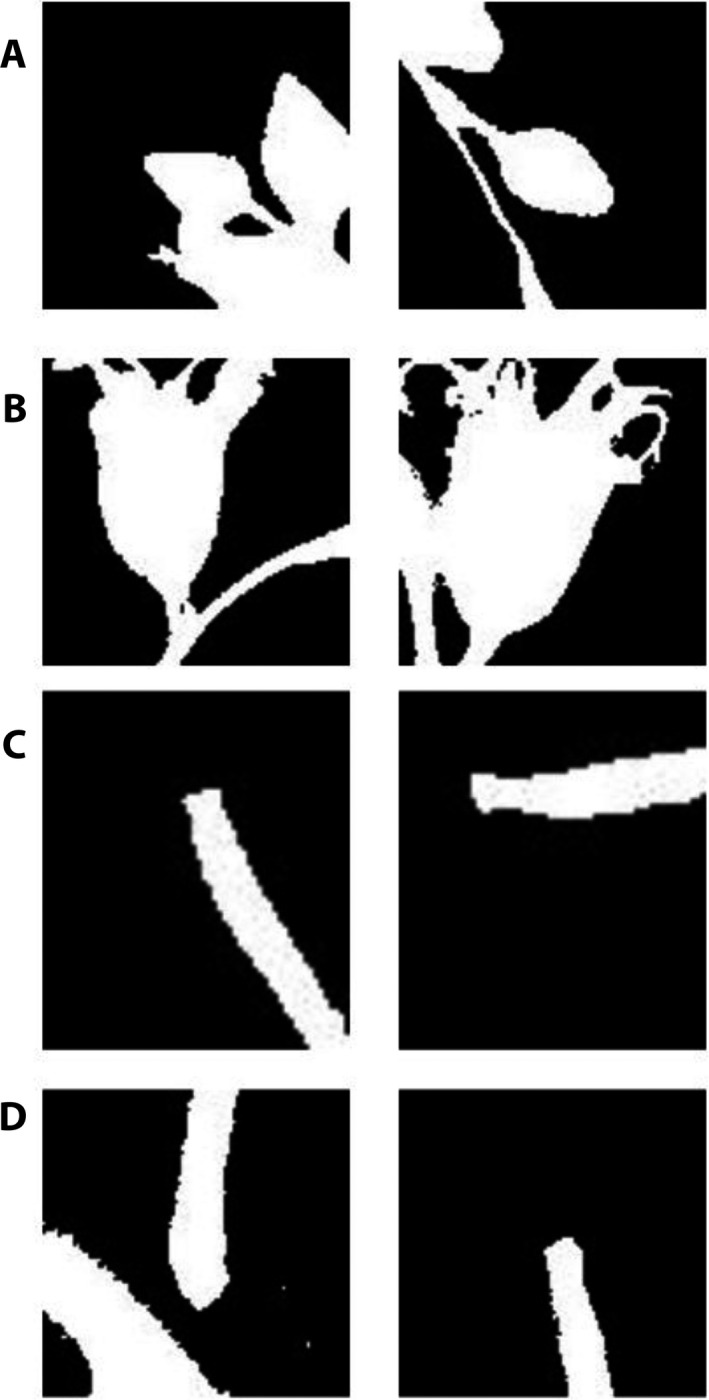
Illustration of two different partial masks for each reproductive structure type in black and white, based on the Otsu segmentation method, automatically generated from points masks. (A) Buds. (B) Flowers. (C) Immature Fruits. (D) Mature Fruits.

The second training set (“*PartialMask*”) is an extension of the Points training data set and includes the partial segmentation of reproductive structures. We applied the classical Otsu segmentation method (Otsu, 1979) within each 100 × 100‐pixel box centered on the initial colored markers. The value of 100 pixels corresponds to a real size of 0.84 cm and represents a good compromise to capture small organs such as buds and flowers while avoiding introducing too much background for large organs such as fruits. This allowed us to automatically capture preliminary masks of each annotated reproductive structure (as illustrated in Figs. 1,  2B, and 3).

**FIGURE 4:**
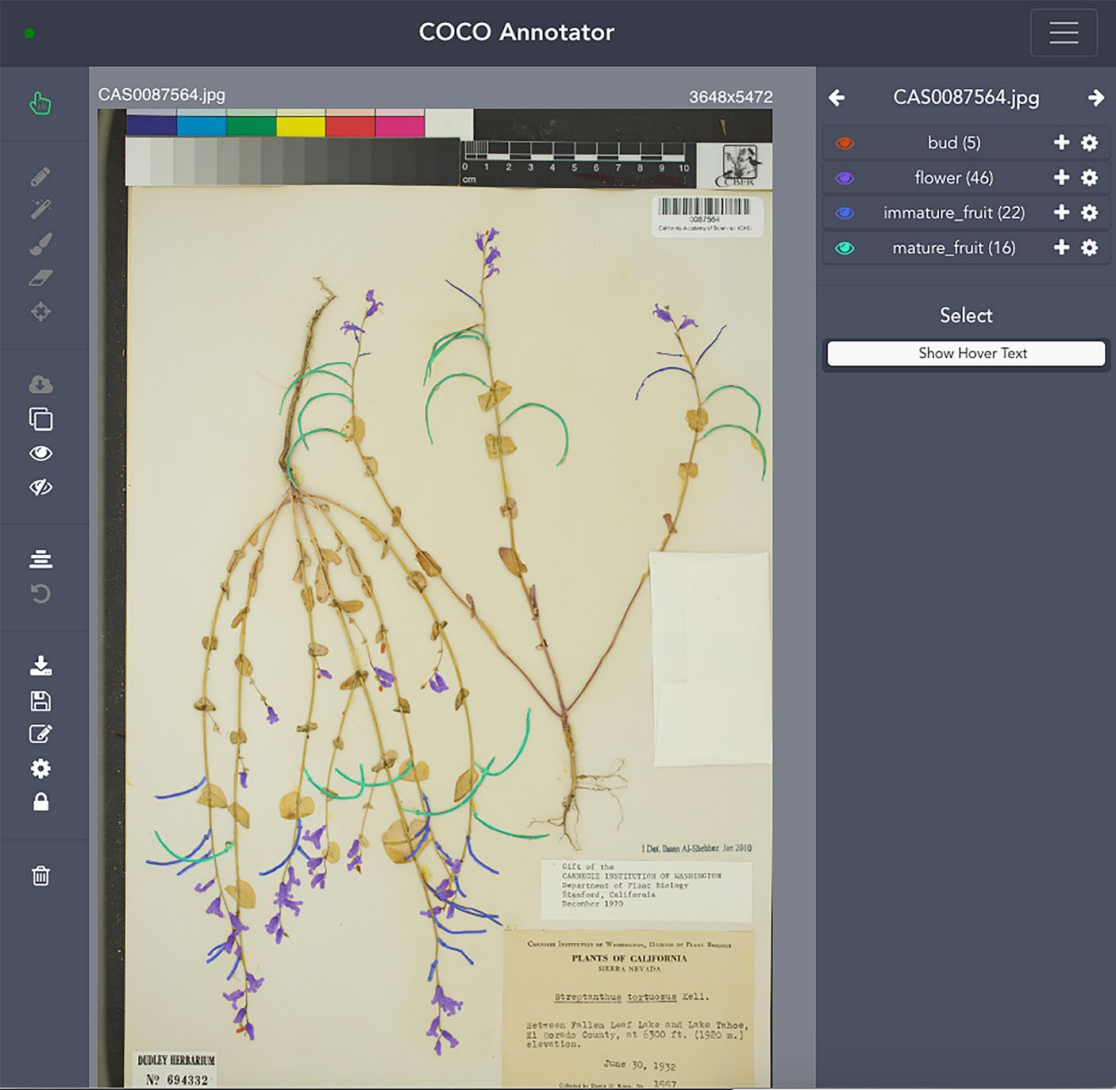
COCO Annotator interface in which a training image of a herbarium specimen has been fully annotated. Five buds (orange), four flowers (purple), 22 immature fruits (blue), and 16 mature fruits (green) have been manually segmented. COCO Annotator is available from https://github.com/jsbroks/coco‐annotator.

The third training set (“*FullMask*”) (illustrated in Figs. 1,  2C) contains only manually annotated masks and was much more time consuming to create. This data set, produced by two of the coauthors (H.G. and P.B.), was cross validated between these two people. We uploaded entire images of herbarium specimens and their partial masks into COCO Annotator (https://github.com/jsbroks/coco‐annotator) (Fig. 4), a web‐based tool for object segmentation, in order to manually draw the full outline of each reproductive structure. This allowed us to capture the full shape of each reproductive organ identified on each specimen. When structures overlapped on the specimen, only the structure in the foreground was annotated, resulting in the exclusion of background structures from the segmentation in that part of the image. A total of 1036 reproductive structures from 21 herbarium specimens were annotated. Details for each reproductive structure are provided in Table 1.

**Table 1:** Number of reproductive structures per data set used in this study.

Reproductive structure type	Training data set	Test set	Total
Flower buds	279	168	447
Flowers	349	299	648
Immature fruits	196	110	306
Mature fruits	212	101	313
Total	1036	678	1714

The test set contains 10 images annotated with the same methodology used for the FullMask training set, thus totaling 678 reproductive structures (see Table 1 for details). It is difficult to precisely indicate the annotation effort in terms of duration, but we estimated that, on average, it took one minute to edit a mask and the label of a reproductive structure. Figure 1C illustrates the precision needed to produce these FullMask training data for the four studied reproductive structures. It is important to remember that these illustrations were chosen for their clarity, but individual reproductive structures are not always so clearly visible (e.g., when structures overlap or have deteriorated), which greatly increases the time required to annotate each specimen. Considering an average of 60 reproductive structures per image, and that an average of one hour was required to annotate each herbarium specimen image, about 30 hours were spent annotating the 31 images of the training and test sets.

### Evaluated deep learning framework

Our fine‐grained detection method is based on the mask R‐CNN architecture (He et al., 2017), which was chosen for its robustness and demonstrated efficiency in instance segmentation tasks and challenges such as MS COCO (Microsoft Common Objects in Context; Lin et al., 2014). We used Facebook’s mask R‐CNN benchmark (Massa and Girshick, 2018) implemented with Pytorch (Paszke et al., 2017). We chose ResNet‐50 as the backbone CNN and the Feature Pyramid Networks (Lin et al., 2017) for instance segmentation. The selection of the number of training epochs was made based on the empirical observation of the model’s training performance. A detailed description of the hyperparameters that were used to train the model is provided in Appendix 1.

### Description of experiments

Based on the predicted and expected reproductive structures of the images in the test set, we computed the following measurements:

#### Counting precision

This metric aims to evaluate the ability of the proposed approach to automatically count the number of reproductive structures present on herbarium sheets. It is defined for a given sheet and type of reproductive structure as the ratio between the number of manually annotated instances (considered to be the “true count”) and the number of automatically detected ones. A counting precision (CP) higher than 100% means that the automated count is overestimated. A CP lower than 100% means that the automated count is underestimated. Complementary to the four types of reproductive structure categories (Buds, Flowers, Immature Fruits, and Mature Fruits), two additional metacategories were considered for performing a secondary CP metric: Buds_Flowers (the sum of the numbers of Buds and Flowers) and Fruits (the sum of the numbers of Developing Fruits and Mature Fruits).

#### Average precision at fixed intersection over union value

**FIGURE 5:**
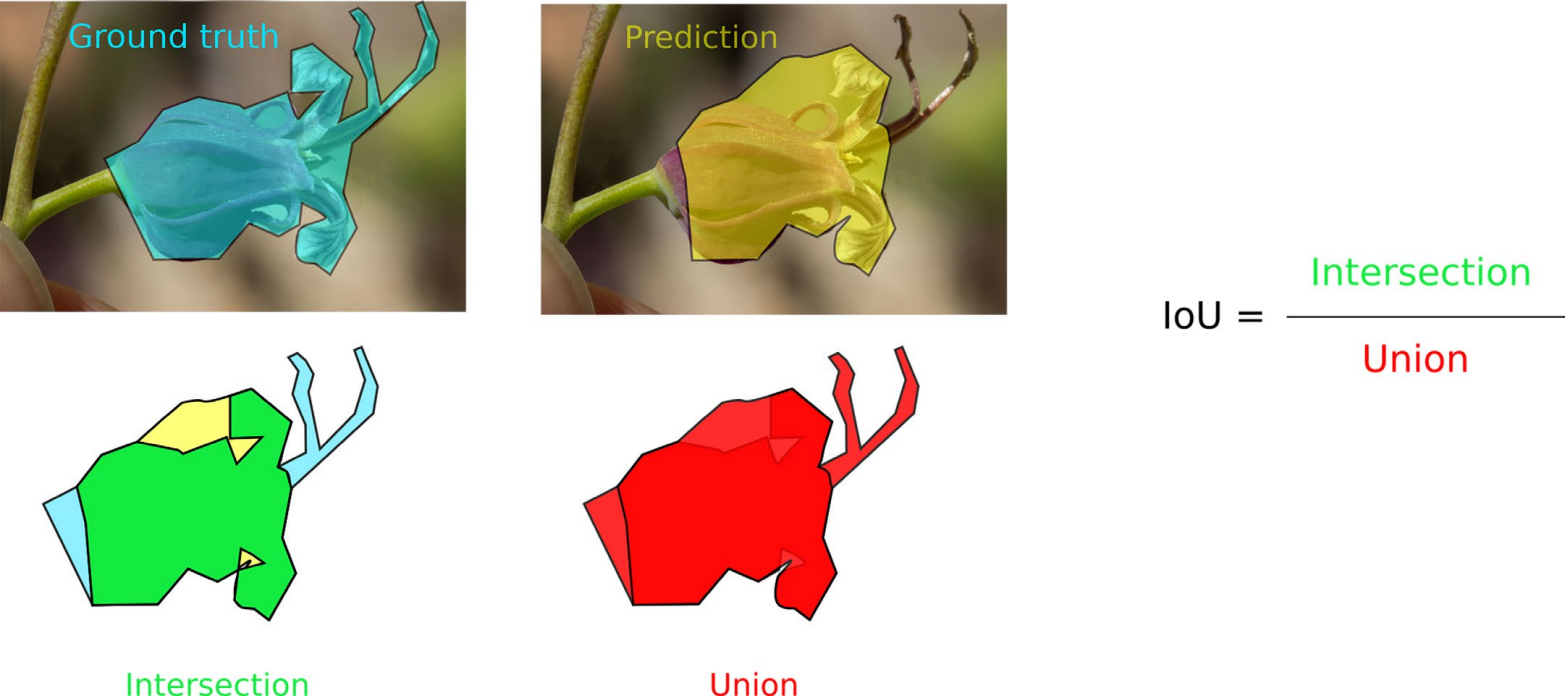
Illustration of the masks used to compute the intersection over union (IoU). The mask of the ground truth (blue) and the mask of a prediction (yellow) are displayed for an image of a *Streptanthus tortuosus* flower (photograph taken by Donna Pomeroy, used under a CC‐BY‐NC license). The mask of the intersection is shown in green, and the one of the union is displayed in red. In this study, only the predicted masks with an IoU value above a fixed threshold value of 50% were used in the final analysis.

This is a common metric used to evaluate the accuracy of instance segmentation tasks, particularly in the context of the popular MS COCO challenge (http://cocodataset.org/#detection‐eval) (Lin et al., 2014). First, all the candidate detections that have sufficient overlap with each object in the ground truth are determined. This is done by computing the union and intersection of the object’s masks (actual and predicted) and retaining only the predicted masks that have an *intersection over union* (IoU) value above a fixed threshold value (in our case, IoU > 50%). A visual illustration of the IoU for a flower of *Streptanthus tortuosus* is provided in Fig. 5. Second, for a given class (i.e., a given type of reproductive structure in our case), all of the remaining matches are sorted by decreasing confidence score of the prediction (i.e., by the maximum probability of the softmax output of the classifier). Finally, the average precision (AP) is computed from that sorted list as follows:

$$AP=\frac{\sum_{k=1}^{n}P\left(k\right)\delta \left(\hat{y}k=yk\right)}{N_{\mathrm{gt}}}$$

where *N_gt_* is the number of object instances in the ground truth, δ(·) is an indicator function equal to 1 if the predicted label of the detected object is equal to the ground‐truth label, and *P(k)* is the precision measured over the top‐*k* results (i.e., the number of correct matches in the *k* first detections divided by *k*).

#### Size‐wise AP

This is the AP computed on instances categorized by their size: (i) Small (1–64 pixels maximum bounding box size), (ii) Medium (65–128 pixels maximum bounding box size), and (iii) Large (>128 pixels). In centimeters, this corresponds to 0.01–0.54 cm for Small, 0.55–1.08 cm for Medium, and >1.08 cm for Large.

#### Detection and confusion probability matrix

This matrix gives the probability of detecting a mask of a particular reproductive structure and the probability of misclassifying it as another reproductive structure. It was computed based on the best match of each mask with respect to its prediction score (i.e., the softmax output).

#### Measurement statistics

Box plots are used to graphically represent the length of the reproductive structures to assess the ability of the proposed approach to automatically estimate the size of reproductive structures in the herbarium sheets. The size of each instance is defined as the diagonal length of the bounding box surrounding the mask of the instance. We considered the same types of reproductive structures as for the CP metric.

#### Measurement precision

This quantitative metric aims to evaluate the ability of the proposed approach to automatically estimate the size of reproductive structures in the herbarium sheets. For a given sheet and type of reproductive structure, the measurement precision is defined as the ratio between the average size of the manually annotated instances in the sheet and the average size of the automatically detected ones. The size of each instance is defined as the diagonal length of the bounding box surrounding the mask of the instance. A measurement precision higher than 100% means the true average size is overestimated. A measurement precision lower than 100% means the true average size is underestimated. We considered the same types of reproductive structures as for the CP metric.

## RESULTS

### Comparison of the three models for counting reproductive structures

**FIGURE 6:**
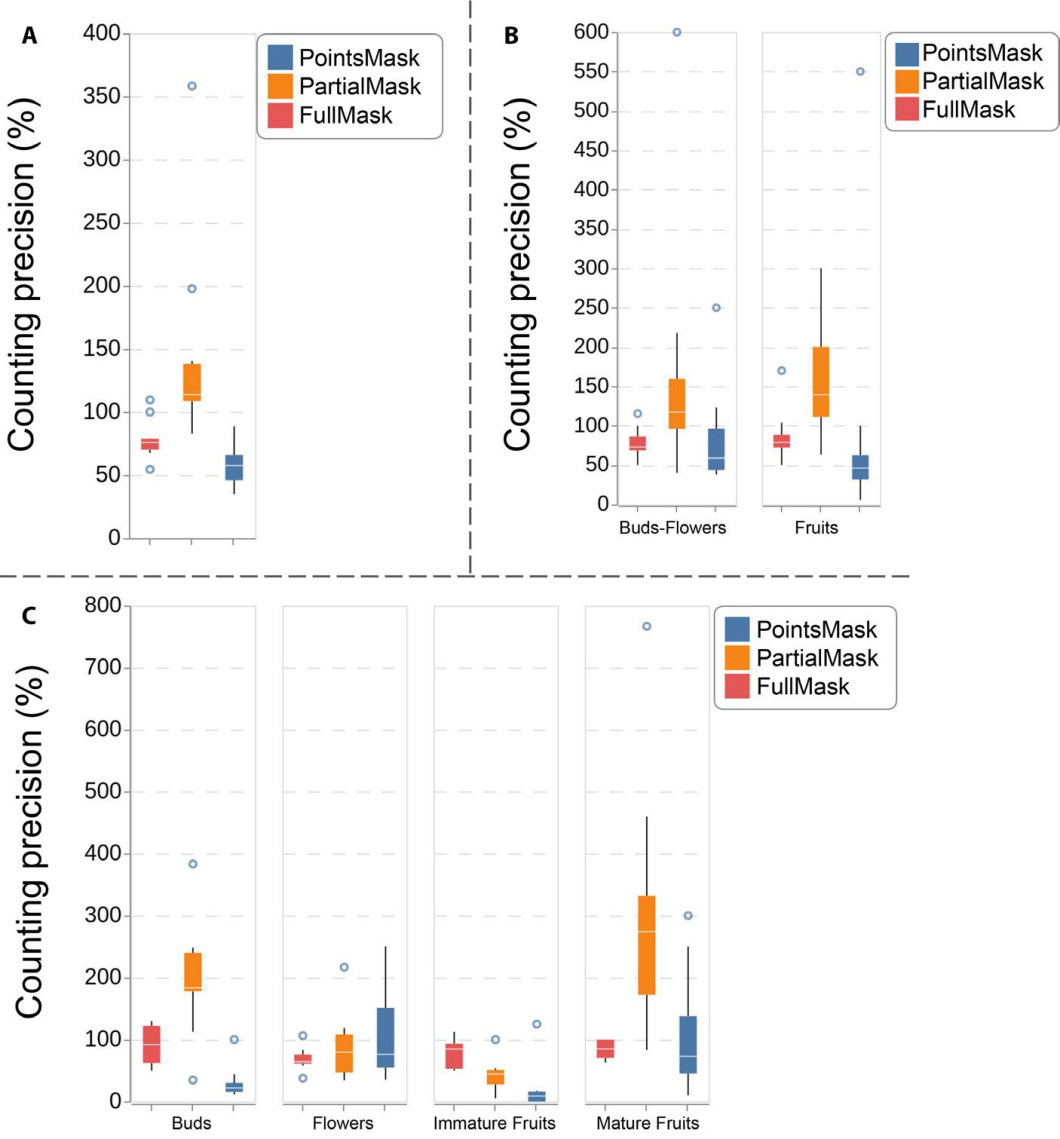
Results of the counting precision for the three learned models. (A) Boxplot of the counting precision across all reproductive structures. (B) Boxplot of the counting precision for the Buds_Flowers and Fruits categories. (C) Boxplot of the counting precision for each reproductive structure type (Buds, Flowers, Immature Fruits, and Mature Fruits).

The comparisons of the CP achieved by the three learned models are presented in Fig. 6 for, respectively, all reproductive structures (Fig. 6A), the Buds_Flowers and Fruits categories (Fig. 6B), and each reproductive structure separately (Fig. 6C). The model with the best CP on average was the R‐CNN‐FullMask, i.e., the model that was trained on the full masks drawn manually. Its average CP was equal to 77.9% of the true number of reproductive structures whereas the CPs of the R‐CNN‐PartialMask and R‐CNN‐PointsMask models were 125.1% and 55.6%, respectively. In addition to these average values, Fig. 6A shows that the CP of the R‐CNN‐FullMask model was much more stable than the other models. It tended to underestimate the counts, but always by the same proportion. Thus, it would be very easy to correct this bias by applying a multiplicative calibration factor. On the contrary, the variance of the CP of the two other models is much higher, thus even a calibration would not be sufficient. Figure 6B shows that the R‐CNN‐FullMask CP is more stable across the Fruits and the Buds_Flowers categories. In contrast, the two other models behave differently for each category and have a much higher variance.

The results obtained for each reproductive structure (Fig. 6C) confirm this trend, but also show that the performance of the R‐CNN‐FullMask differed among the different types of reproductive structures. On average, it was less biased when counting Immature Fruits (average CP = 95.5%) and Buds (average CP = 86.3%), but in terms of stability, it performed better on Flowers and Mature Fruits. Appendix S1 provides details of the ground truth and the obtained results of the three learned models for the count of each reproductive structure, in each image of the test set.

### Detailed analysis of the detection performance of the R‐CNN‐FullMask model

**FIGURE 7:**
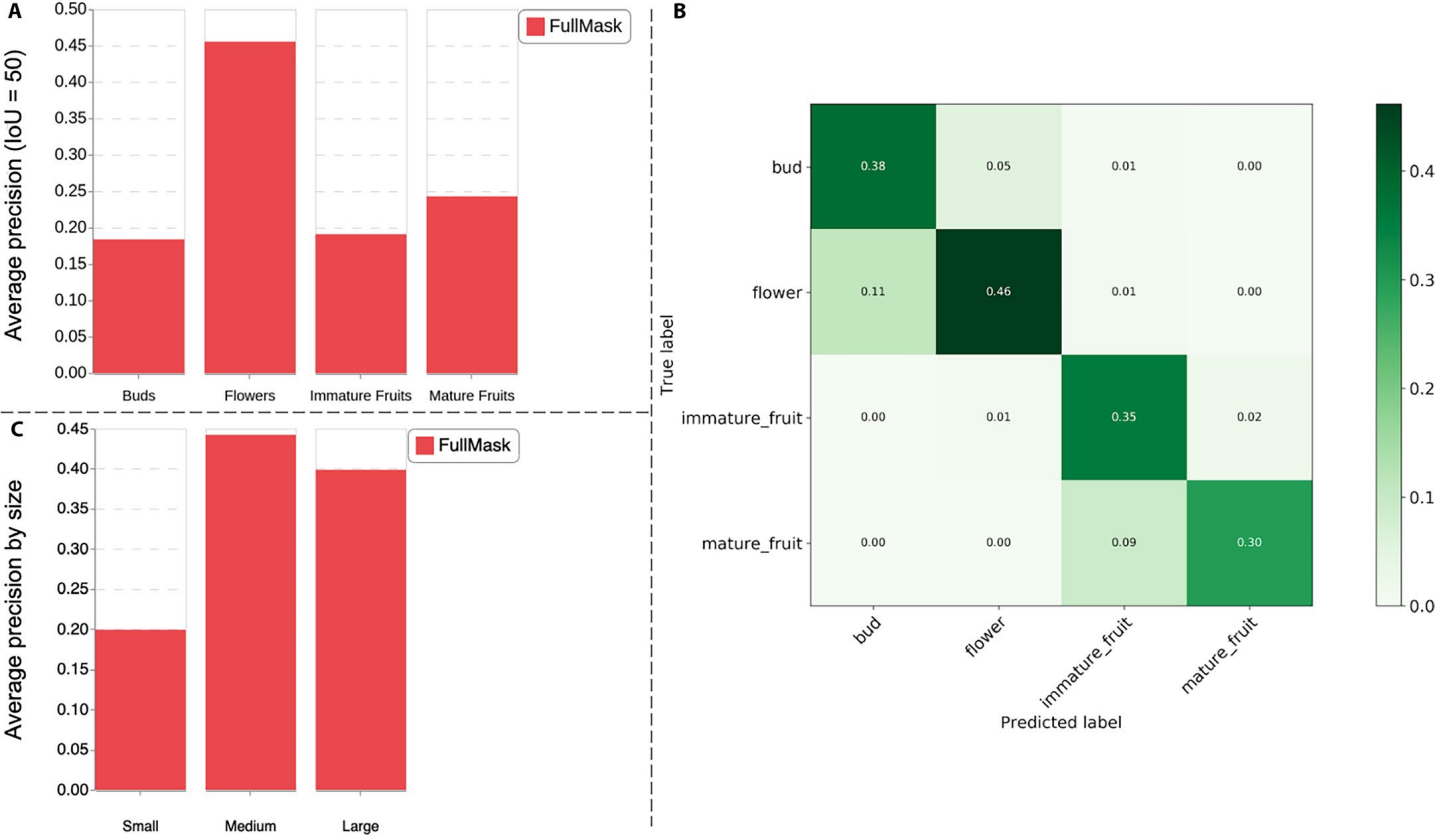
Detection performance of the R‐CNN‐FullMask model. (A) Average precision (intersection over union [IoU] = 50%) of the R‐CNN‐FullMask model for each reproductive structure. (B) Confusion matrix of the R‐CNN‐FullMask model (i.e., the probability of detecting the incorrect organ type). The row probabilities do not sum to 1 because an organ may not be detected at all; (1 – sum(row)) gives the probability of misdetection. (C) Average precision (IoU = 50%) of the R‐CNN‐FullMask model for each object size category: Small, 1–64 pixels; Medium, 65–128 pixels; Large, >128 pixels.

Given that the R‐CNN‐FullMask model exhibited the most accurate CP, we conducted a more detailed analysis of its detection performance. In particular, Fig. 7A reports its AP for detecting each type of reproductive structure at IoU = 50% and Fig. 7B displays the detection and confusion probability matrix. Both figures show that the type of reproductive structure most accurately detected was Flowers, followed by Mature Fruits, Immature Fruits, and Buds. This is consistent with the CP experiment, in which the most stable counts were achieved for Flowers. Not all flowers were detected, but the percentage of detected ones is quite stable across all herbarium specimens so that a multiplicative calibration factor could be used to generate accurate counts. On the contrary, Buds, Immature Fruits, and Mature Fruits seem to be more difficult to detect, mainly because of a high rate of misdetection and false alarms (misclassifications are relatively rare, so they cannot explain the low AP values).

Figure 7C displays the size‐wise AP (still at IoU = 50%), revealing that the size of the reproductive structures has a strong impact on performance. Reproductive structures more than 1.52 cm long (i.e., the Large category in Fig. 7C) were detected with an AP of 0.4, while structures less than 0.76 cm long were detected with an AP of 0.2; the highest value of AP (0.44) was obtained for medium‐sized structures. It is important to remember that the hyperparameters of our model have been chosen in such a way as to cover all sizes of the objects present. The higher probability of misdetection of the smallest objects is thus likely to be due to a bias rather than a problem of resolution. This will have to be explored more deeply in further work.

### Evaluating the performance of the R‐CNN‐FullMask model for measuring the size of reproductive structures

**FIGURE 8:**
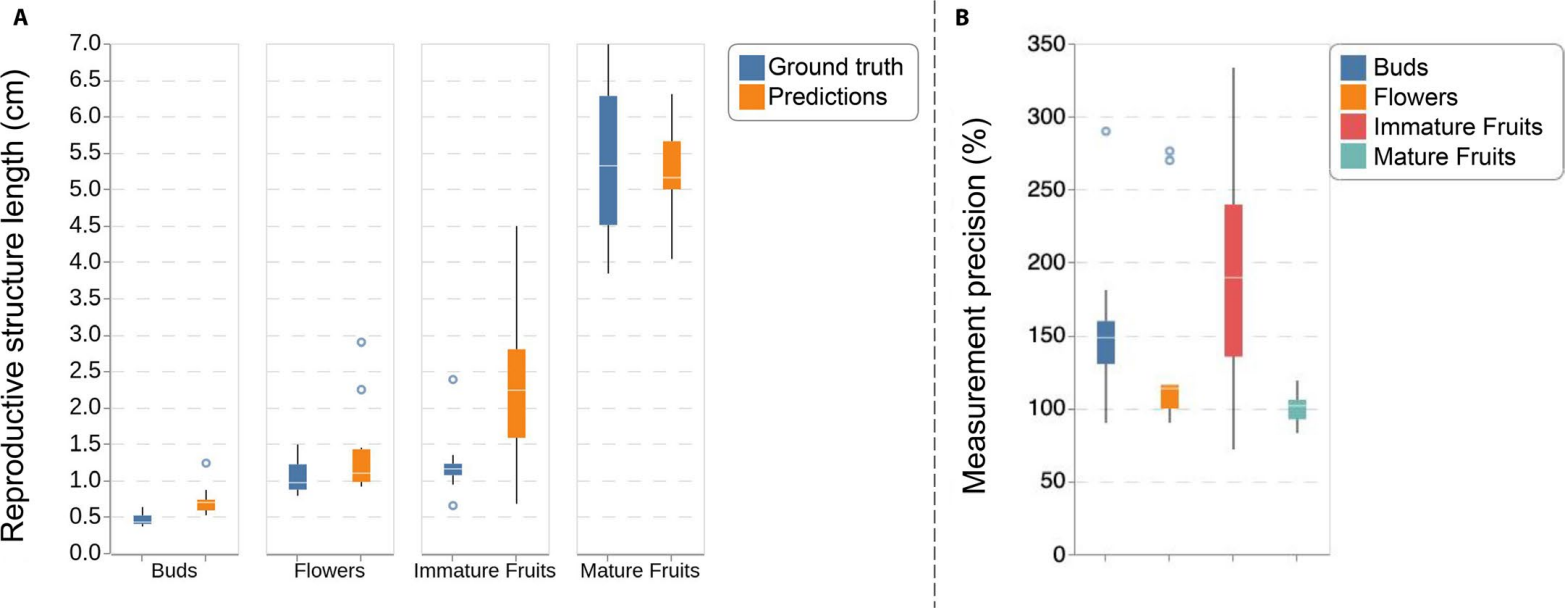
Performance of the R‐CNN‐FullMask model for measuring the size of reproductive structures. (A) Measurement statistics comparing the ground truth and the predictions of the R‐CNN‐FullMask with respect to the size (in centimeters) of the reproductive structures estimated through the diagonal of the bounding boxes. (B) Measurement precision per object of the R‐CNN‐FullMask of the reproductive structures estimated through the diagonal of the bounding boxes.

Figure 8A shows the boxplot of the true and estimated object’s size for each type of reproductive structure. This figure displays the boxplot of the Measurement statistics and shows that quite accurate measurements can be achieved for most reproductive structures, except in the case of immature fruits, which remain very difficult to detect and segment accurately. Indeed, the variance of the prediction for immature fruits is the highest among all reproductive structures, particularly for those that are longer than 3 cm, probably because their visual appearance is very similar to that of mature fruits. Figure 8B shows the Measurement Precision and highlights the overestimation of sizes for Immature Fruits and, to a lesser extent, for Buds, while the variance is relatively low for Mature Fruits and Flowers. Mature fruits in particular would be the best reproductive structures to automatically detect in a larger data set using the current R‐CNN‐FullMask model for conducting phenological studies based on fruit size and the date of maturity.

## DISCUSSION

The purpose of this work was to develop and test an instance segmentation method for the fine‐scaled detection of different reproductive structures in herbarium specimens. In particular, the aim was to evaluate the performances of models based on different types of training data. For the best of these models (i.e., R‐CNN‐FullMask), we further aimed to precisely analyze its potential for counting, detecting, and measuring reproductive structures in order to facilitate new ways to conduct phenological studies on natural history collections.

One main outcome of this study is that deep learning technologies such as mask R‐CNN models are highly influenced by (i) the type of training data on which they are developed, (ii) the type of reproductive structure targeted, and (iii) organ size. Indeed, although points masks are the most quickly produced by annotators compared with partial masks or full manual masks, they are much less efficient for the automated counting of reproductive structures. Nevertheless, our study has shown that points masks can be useful to produce partial masks based on an automated segmentation approach within a window generated around each point, which can significantly increase the performance of models trained from these raw data. Full masks clearly provide the most efficient training data, as they can capture the full visual information associated with all the reproductive structures present on several different herbarium specimens.

This study has clearly shown that a mask R‐CNN based on full masks has the potential to reliably contribute to the detection, counting, and measurement of some of the reproductive structures targeted. Flowers seem to be the most accurately captured, as (i) they are much larger than buds, (ii) they are more isolated than buds, which are often aggregated in a cluster at the tip of inflorescences, and (iii) they overlap less with other plant structures in *Streptanthus tortuosu* *s*, in contrast to its frequently overlapping long thin fruits. This is an important result, as we know from the cross‐herbaria analysis of Lorieul et al. (2019) that herbarium specimens are often collected in flower; for example, among the specimens from the New England Vascular Plant collection (https://www.idigbio.org/wiki/index.php/Mobilizing_New_England_Vascular_Plant_Specimen_Data) and Florida State University’s Robert K. Godfrey Herbarium (Tallahassee, Florida, USA) used in Lorieul et al.’s (2019) study, 64.9% and 73.9% bore flowers, respectively.

The influence of organ size on the automated detection and identification of reproductive structures is a key issue to take into account; there is a real need to improve the analysis of small structures in such visual collections. A potential solution could be to work with higher‐resolution images, which was not possible in our study due to computational resource limitations. The impact of organ size identified here (i.e., the lowest AP value obtained for small structures) is important as it indicates that this approach will be more efficient on taxa bearing relatively large reproductive structures. Clades characterized by such traits (e.g., large‐flowered and large‐fruited Fabaceae, Onagraceae, Brassicaceae) could be much more suitable for this type of analysis. Similarly, taxa with loosely arranged inflorescences or singly borne flowers (instead of dense ones) will be relatively easier to analyze.

Other key issues are the capacity to take into account the high variability of some of these reproductive structures, such as immature fruits, which are highly diverse in terms of size, shape, color, and texture. Potential improvements to address these constraints could include the use of much larger training data sets, notably in terms of the number of each type of reproductive structure. This could allow such training data sets to sample a much larger diversity of development stages, from closed buds to ripe fruits, with greater intensity. It is important to recall that the performance metrics reported here are based on a rather small training data set comprising 21 herbarium specimens. Thus, the performance could be considerably enhanced by enriching the data set, for example with specimens from different ecosystems, collected at different periods of the year, and conserved for different durations. In particular, this could potentially improve our model’s robustness and generalization capability, thereby allowing its use with other *Streptanthus* species. The use of complementary training data representing other species or structures (such as leaf or stem masks) could also be beneficial for the transfer of learning strategies that have already proved their efficiency for other herbarium visual classification tasks (Carranza‐Rojas et al., 2017).

Other computation techniques could also contribute to the improvement of model performance. Indeed, the masks themselves are computed by thresholding a local heatmap, an image whose intensity is proportional to the likelihood that the pixel belongs to the detected object. This thresholding of the heatmap can be seen as a loss of information, and it could be much more efficient to optimize the treatment directly from the set of heatmaps rather than the set of masks. This could be used, for example, to select masks with a very high score of confidence for measurement analysis, instead of trying to measure all detected structures, even those with low confidence scores. Model combination is also a promising solution that has yielded performance improvement for other visual biological classification tasks (as in the study of Goëau et al., 2017). This is a computation method that consists of training multiple models instead of a single model and then combining the predictions generated by these models. This method, also called “ensemble learning,” can be done with many different computational and statistical strategies and usually improves results by generating predictions that are more accurate than those produced by any single model.

We chose to evaluate the use of the mask R‐CNN approach, already used in other biological contexts (e.g., Champ et al., 2020), as it has the advantage of allowing the detection, counting, and potential measurement of detected structures within a single model. Other approaches could be evaluated, such as the strategy presented by Chattopadhyay et al. (2017), where the counting task is divided into easier and smaller object detection tasks on subsections of images. DeepSetNet (Rezatofighi et al., 2017) is an alternative technique that simultaneously detects and counts objects trained with the labels of the true count. Seguı et al. (2015) trained models using only the true count on the global image. Some of the most popular approaches are based on a “density” method (Arteta et al., 2016; Boominathan et al., 2016). In this method, models are trained on the annotation of object centers, on which a density map is then computed and integrated to obtain a count value. All of these approaches could greatly enrich our capacity to report the presence and abundance of reproductive structures in herbarium collections, and without doubt could contribute to enlarging the huge research potential of these invaluable collections. Combined with other machine learning techniques (such as in the studies of Meineke et al., 2020; Ott et al., 2020; Pryer et al., 2020; Weaver et al., 2020; White et al., 2020), they could largely increase the benefits of using the newly digitized herbarium data sets.

## Supporting information


**APPENDIX S1.** Detail of the ground truth and results for each image of the test set, showing ground truth (in light blue) and the three learned models (dark blue = R‐CNN‐PointsMask, orange = R‐CNN‐PartialMask, and red = R‐CNN‐FullMask) for the count of buds (A), flowers (B), immature fruits (C), and mature fruits (D) of each image of the test set. The *x*‐axis titles indicate the name of the images in the test set, while the *y*‐axis ordinates denote the number of reproductive structures. The predictions of the R‐CNN‐FullMask are often closest to the ground truth.

## Data Availability

Data used and produced for this study are accessible on Zenodo (Goëau et al., 2020), a free and open platform for preserving and sharing research output.

## APPENDIX 1 Detailed description of the hyperparameters used to train the mask R‐CNN architecture.

### Input image size

—Images are resized such that the longest edge is 2048 pixels and the shorter one is 1024 pixels, in order to have sufficient pixels related to small objects such as the buds (by default the mask R‐CNN implementation used here considers a longest edge of 1024 pixels and a shorter one of 600 pixels).

### Anchor size and stride

—Anchors are the raw regions of interest used by the *region proposal network* to select the candidate bounding boxes for object detection. We chose their size to guarantee that the targeted objects have their size covered. The anchor size values were set to [32;64;128;256;512], the anchor stride values to [4;8;16;32;64], and the anchor ratios to [0.5;1;2].

### Non‐maximal suppression

—The non‐maximal suppression (NMS) quantifies the degree of overlap tolerated between two distinct objects (set to 0.5, the default value in the mask R‐CNN implementation used here).

The training of the model was run on an NVidia Geforce RTX 2080 Ti (NVidia Corporation, Santa Clara, California, USA) using stochastic gradient descent with the following parameters: batch size of 3, total number of epochs set to 10,000, and learning rate of 0.01. We trained our model for several periods of time and stopped training when the loss converged or stopped decreasing. More specifically, we use a technique called the “Panda approach,” in which all training parameter choices were made on the basis of the empirical observation of the model’s training performance. We use a *warmup strategy*, where the learning rate increases linearly from 0.005 to 0.01 during the first five epochs. To improve robustness and minimize the variance of the model, we applied a large set of data augmentation techniques, including random horizontal, random rotations, and random variations on color contrast, saturation, brightness, and hue color values.
