# Supplementary material for: A new fine‐grained method for automated visual analysis of herbarium specimens: A case study for phenological data extraction
Source: Appl Plant Sci. 2020 Jul 1;8(6):e11368. doi: 10.1002/aps3.11368 (PMC7328656; doi:10.1002/aps3.11368)

doi: 10.1002/aps3.11368

APPENDIX S1. Detail of the ground truth and results for each image of the test set, showing ground truth (in light blue) and the three learned models (dark blue = R-CNN-PointsMask, orange = R-CNN-PartialMask, and red = R-CNN-FullMask) for the count of Buds (A), Flowers (B), Immature Fruits (C), and Mature Fruits (D) of each image of the test set. The *x*-axis titles indicate the name of the images in the test set, while the *y*-axis ordinates denote the number of reproductive structures. The predictions of the R-CNN-FullMask are often closest to the ground truth.

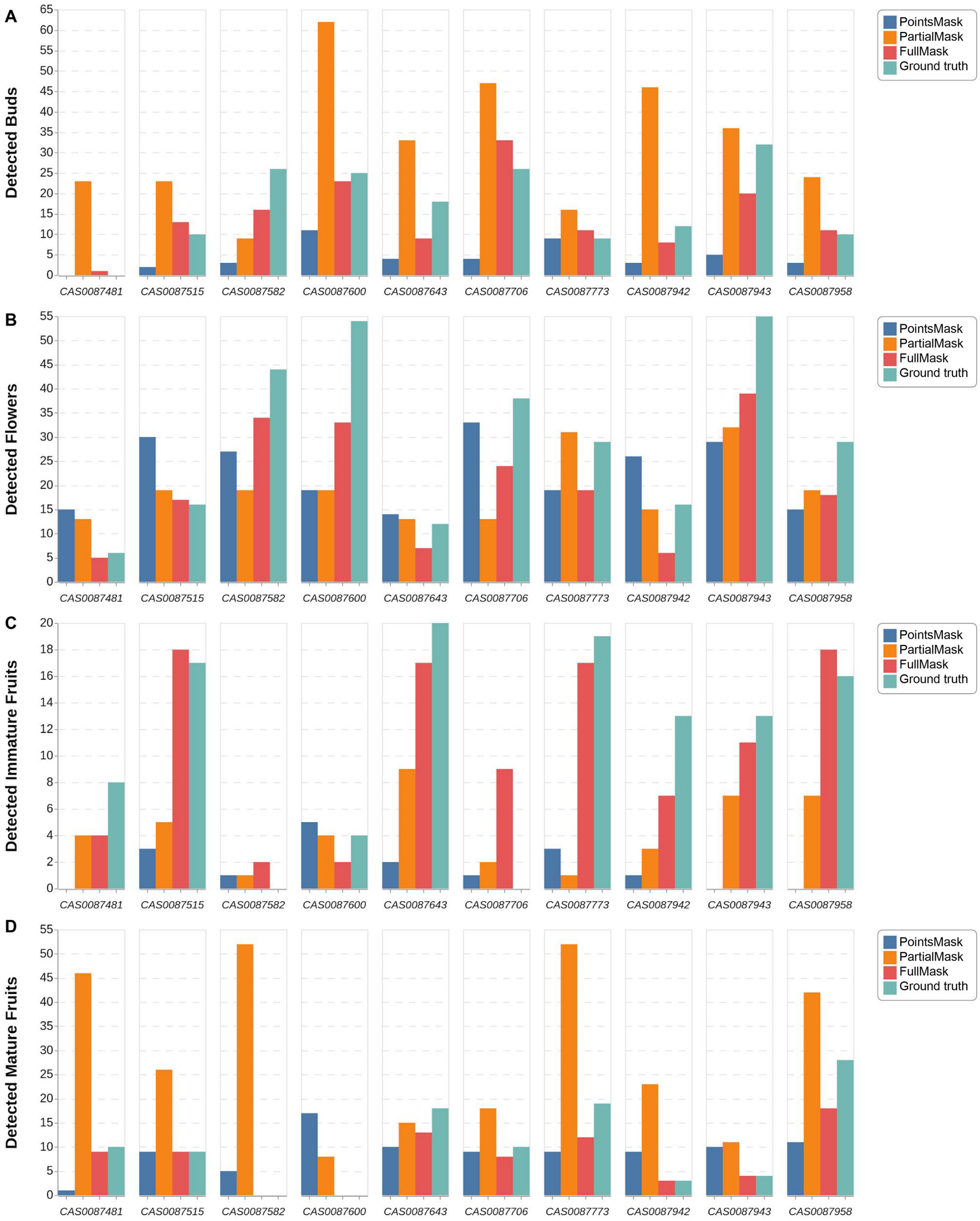

Supplement: Supplementary file 1 — APPENDIX S1. Detail of the ground truth and results for each image of the test set, showing ground truth (in light blue) and the three learned models (dark blue = R‐CNN‐PointsMask, orange = R‐CNN‐PartialMask, and red = R‐CNN‐FullMask) for the count of buds (A), flowers (B), immature fruits (C), and mature fruits (D) of each image of the test set. The x‐axis titles indicate the name of the images in the test set, while the y‐axis ordinates denote the number of reproductive structures. The predictions of the R‐CNN‐FullMask are often closest to the ground truth. [file APS3-8-e11368-s001.pdf]
